# Regulatory factor X‐5/SCL/TAL1 interruption site axis promotes aerobic glycolysis and hepatocellular carcinoma cell stemness

**DOI:** 10.1002/kjm2.12922

**Published:** 2024-12-24

**Authors:** Zhi‐Zhong Zhang, Zi‐Ming Wang, Hao‐Wen Zhang, Yan‐Xin Gong, Hao‐Ran Sun, Wei Zhang

**Affiliations:** ^1^ Department of General Surgery Ward One Anyang Tumor Hospital Anyang Henan China; ^2^ Pathological Center Anyang Tumor Hospital Anyang Henan China

**Keywords:** glycolysis, hepatocellular carcinoma, RFX5, stemness, STIL

## Abstract

The incidence and development of various tumors, such as hepatocellular carcinoma (HCC), are linked to tumor stem cells. Although research has revealed how important SCL/TAL1 interruption site (STIL) is in many human tumors, the impact of STIL on HCC stem cells is poorly understood. This study aimed to examine the regulatory mechanisms and the function of STIL in the stemness of HCC tumor cells. Bioinformatics analysis was applied to determine the *STIL* and regulatory factor X‐5 (*RFX5)* expression in HCC tissues. Immunohistochemistry (IHC) was used to detect the expression of STIL and RFX5 in HCC tissues. Quantitative real‐time polymerase chain reaction was utilized to measure the *STIL* and *RFX5* expression levels in HCC cells. The viability of the cells was assessed by the Cell Counting Kit‐8 assay. The sphere formation assay was used to evaluate the sphere‐forming capacity. The expression levels of the stem cell markers SOX2, Oct‐4, CD133, CD44, the glycolysis‐related proteins LDHA, HK2, AKT, p‐AKT, and β‐catenin were assessed by Western blot. Lactate production, oxygen consumption rate, and extracellular acidification rate were measured to assess the glycolytic capacity of HCC cells. Chromatin immunoprecipitation and dual‐luciferase experiments were performed to validate the connection between RFX5 and STIL. Bioinformatics analysis determined that *STIL* exhibited high expression in HCC tissues and was enriched in the glycolysis pathway. In addition, the expression of glycolysis marker genes was positively correlated with *STIL* expression. Cell experiments verified that the activation of the glycolysis pathway by overexpression of *STIL* promoted stemness in HCC. Molecular experiments also revealed the binding relationship between *STIL* and *RFX5*. IHC detected high expression of STIL and RFX5 in HCC tissues. Cell functional experiments revealed that RFX5 could influence the HCC cells stemness by activating the STIL transcription via the glycolysis pathway. This study identified a novel role for the RFX5/STIL axis in HCC progression, which may offer treatment targets for HCC.

## 1 INTRODUCTION

Being the sixth most prevalent cancer globally, the incidence of hepatocellular carcinoma (HCC) is increasing.^1^ Liver transplantation or surgical resection may be beneficial for patients with early‐stage HCC; however, radical treatment is not practical for patients diagnosed with late‐stage HCC, and systemic therapy is the only feasible option for them.^2^ HCC shows notable resistance to conventional radiotherapy and chemotherapy.^3^ Thus, to develop novel treatment approaches, it is crucial to understand the functional mechanisms underlying the growth and progression of HCC.

Cancer stem cells (CSCs) are self‐renewing cell types found in most cancer types. They promote tumor occurrence, recurrence, proliferation, metastasis, and drug resistance after treatment.^4^ The stemness of tumor cells is influenced by a wide range of factors. For example, RALYL can bind to *TGF‐β2* mRNA, decrease the m6A modification of *TGF‐β2* mRNA, increase the stability of *TGF‐β2* mRNA, and upregulate TGF‐β2 to promote the stemness of HCC cells.^5^ Wang et al.^6^ verified that CRIP1 can interact with E3 ligase STUB1 and BBOX1 to facilitate BBOX1 ubiquitination and proteasome degradation, causing the carnitine downregulation and stimulation of HCC cell stemness. Moreover, glycolysis may have an impact on CSCs. Glycolysis was promoted by histone deacetylase 11 through the inhibition of LKB1 expression in HCC, thereby preventing the AMP‐activated protein kinase (AMPK) signaling pathway and enhancing cancer stemness.^7^ Chen et al.^8^ confirmed that HBV x protein can induce BNIP3L‐dependent mitophagy to promote the glycolytic metabolic pathway, thereby enhancing the cell stemness of HCC. Although increasing evidence revealed potential mechanisms affecting cancer cell stemness, the relevant molecular mechanisms regulating HCC cell stemness are not yet fully understood. This study further investigated the regulatory mechanism of HCC cell stemness.

SCL/TAL1 interruption site (*STIL*) is a cilia‐related gene, and the formation of primary cilia is intimately linked to STIL expression.^9^ Furthermore, STIL is a cell cycle regulatory protein that is recruited to the centrosome in mitosis to stimulate centriole duplication in mitotic cells.^10^ It has been confirmed that STIL is abnormally overexpressed in various tumors, regulating tumor progression. STIL is highly expressed in HCC tissues, and its high expression is associated with poor patient prognosis.^11^, ^12^ For instance, STIL expression is elevated in tissues from nasopharyngeal carcinomas, and its knockdown can prevent cancer cells from migrating and invading, accelerating the transition from the G1/S phase and apoptosis.^13^ Ji et al.^14^ discovered that osteosarcoma has high expression of STIL. Silencing STIL can decrease the malignant progression of osteosarcoma cells and increase apoptosis. In addition, STIL regulates the stemness of tumor cells. For instance, through the transduction of Wnt and Shh signals, STIL promotes colorectal cancer cell growth and stemness.^15^ The exact mechanism by which STIL contributes to HCC stemness is still unknown despite these findings. Therefore, this study will further explore the expression of STIL in HCC cells to better understand the functional mechanism of STIL in the HCC progression.

In summary, our research offered a new theoretical foundation for the HCC treatment by indicating that regulatory factor X‐5 (RFX5) phosphate buffer saline and STIL might be novel therapeutic targets for HCC.

## 2 MATERIALS AND METHODS

### 2.1 Bioinformatics analysis

The mRNA expression data of HCC were downloaded from The Cancer Genome Atlas (TCGA) database, and differential analysis was performed using edgeR to obtain differentially expressed mRNAs (DEmRNAs). Then, the target gene was determined by bioinformatics data and relevant literature, and its correlation with the stemness index mRNAsi was analyzed. Kaplan–Meier analyzed the correlation between target genes and prognosis in HCC patients. Gene set enrichment analysis and correlation analysis were conducted to study the regulatory mechanism of the target gene. The upstream transcription factors of the target gene were identified using the KnockTF database and were determined using Pearson correlation. The binding sites between the transcription factor and target gene were predicted by the Homo sapiens comprehensive model collection (HOCOMOCO).

### 2.2 Clinical sample collection

In order to investigate the correlation of target genes and their upstream transcription factors with the clinical tissues of HCC patients, we collected five pairs of tumor tissues and their paracancerous tissues of HCC patients from Anyang Tumor Hospital during the period from September 2023 to August 2024, and the experiments related to the collection of the clinical samples were conducted in strict accordance with the requirements of the Medical Ethics Committee of Anyang Tumor Hospital.

### 2.3 Immunohistochemistry

HCC tumor tissue and paracancerous tissue samples collected from the clinic were embedded in paraffin wax and cut into tissue sections of about 5 μm using a microtome. The sections were dewaxed twice for 10 min each time, washed three times with phosphate buffer saline (PBS), then placed in a solution containing 5% skimmed milk for 30 min at room temperature, and endogenous peroxidase activity was blocked with hydrogen peroxide for 10 min at room temperature. The tissue sections were incubated with primary antibodies containing RFX5 and STIL at 4°C overnight. The slices were rinsed with PBS and incubated with secondary antibodies at 37°C for 2 h. The slices were removed, washed with PBS, and color‐developed with diaminobenzidine (DAB). Finally, the sections were stained with hematoxylin for 2 min, and the staining of the tissue sections was observed under a light microscope. Rabbit anti‐human primary antibodies (Anti‐RFX5 and Anti‐STIL) and goat anti‐rabbit immunoglobulin G (IgG) H&L horseradish peroxidase (HRP) were obtained from Abcam (UK).

### 2.4 Cell cultivation

Human embryonic kidney cells 293T, human normal liver cells LX‐2, human liver cancer cell line Hep3B, Huh7, and SKHEP‐1 were all bought from the BeNa Culture Collection (BNCC, China). Huh7, SKHEP‐1, and 293T cells were cultivated in Dulbecco's Modified Eagle's Medium‐High Glucose (DMEM‐H) Dulbecco's Modified Eagle Medium/Nutrient Mixture F‐12 medium containing 1% penicillin/streptomycin (P/S, Gibco) and 10% fetal bovine serum (FBS, Gibco), and 293T cells required extra 2 mM glutamine. Hep3B cells were cultivated in Eagle's Minimal Essential Medium (EMEM) medium containing 1% P/S and 10% FBS, and LX‐2 cells were cultivated in Roswell Park Memorial Institute (RPMI)‐1640 medium containing 1% P/S and 10% FBS. All cells were incubated at 37°C with 5% CO_2_.^16^, ^17^

### 2.5 Cell transfection

sh‐STIL, oe‐STIL, sh‐RFX5, and corresponding negative controls were synthesized by Sangon Biotech (China). Cells were cultivated overnight in a 6‐well plate with about 5 × 10^4^ cells per well and then transfected using Lipofectamine™ 2000 (Invitrogen, USA). After 48 h, cells were collected for further experiments. Regular detection confirmed that the cells were not contaminated with mycoplasma.^18^

### 2.6 Quantitative real‐time polymerase chain reaction

Following the directions, total RNA was extracted from cells using the RNeasy Mini Kit (Qiagen, Germany). Cells were thoroughly homogenized by the 1 mL pipette after being lysed in RLT lysis buffer. RNeasy Mini Kit columns were used to purify the RNA, which was then dissolved in water free of RNase. cDNA was synthesized by PrimeScript™ RT Reagent Kit (Takara, Japan). Subsequently, quantitative real‐time polymerase chain reaction (qRT‐PCR) assay was carried out with TB Green® Premix Ex Taq™ (Takara, Japan) on an Applied Biosystems™ 7500 real‐time PCR system. With *β‐actin* acting as the reference gene, the expression of *RFX5* and *STIL* was examined using the 2^−ΔΔCt^ method. At least three replications of each experiment were conducted. Please refer to Table 1 for the used primers.^19^

**TABLE 1:** Primers used in quantitative real‐time polymerase chain reaction.

Gene	Sequence	
STIL	Forward primer	5′‐CCCAACGCCAACTGGAGATTT‐3′
	Reverse primer	5′‐AGTCGGATGGTCTTCTCAGTC‐3′
RFX5	Forward primer	5′‐CACTGACACCTGTCTGCCAAA‐3′
	Reverse primer	5′‐CCTTCGAGCTTTGATGTCAGGG‐3′
β‐Actin	Forward primer	5′‐GTCTCCTCTGACTTCAACAGCG‐3′
	Reverse primer	5′‐ACCACCCTGTTGCTGTAGCCAA‐3′

Abbreviation: STIL, SCL/TAL1 interruption site.

### 2.7 Cell Counting Kit‐8 assay

First, 2000 cells were seeded into a 96‐well plate with six replicates per group. On the first day, four identical plates were seeded and put in the incubator for cell culture. After culturing for 24, 48, 72, and 96 h respectively, the Cell Counting Kit‐8 (CCK‐8) reagent (10 μL) was added to each well, and the absorbance at 450 nm was measured using the microplate reader after incubation at 37°C for 2 h. GraphPad Prism 8 was used to plot the growth curves of each cell group based on the results of the 4‐day experiment.^20^

### 2.8 Cell sphere formation assay

Cells (5 × 10^3^ cells/well) were seeded in serum‐free Dulbecco's Modified Eagle Medium/Nutrient Mixture F‐12 (DMEM/F‐12) medium containing 2% B27, 20 ng/mL epidermal growth, and 20 ng/mL fibroblast growth factor. After 2–3 weeks of cultivation, the growth of tumor spheres was examined under a microscope, and the quantity of third‐generation tumor spheres was recorded and counted. Each generation was cultured for 14 days.^16^, ^18^

### 2.9 Western blot

HCC cells were harvested after being washed with phosphate‐buffered saline and subjected to sonication at 4°C in radioimmunoprecipitation assay buffer (150 mM NaCl, 50 mM Tris–HCl pH 8.0, 0.1% sodium dodecyl sulfate, 0.5% sodium deoxycholate, and 1% Triton X‐100) supplemented with 1% protease inhibitor cocktail (Thermo Fisher Scientific, USA). Protein concentration was determined using the bicinchoninic acid assay kit (Sigma, USA) after lysate suspension was obtained by centrifuging at 4°C for 10 min. In 5× sample buffer, protein denaturation was carried out for 5 min at 100°C. Equal volumes of protein were loaded onto an sodium dodecyl sulfate‐polyacrylamide gel electrophoresis (SDS–PAGE) gel and subsequently moved to a polyvinylidene fluoride membrane for protein blotting. The membrane was blocked with 5% skim milk at room temperature for 1 h, and then it was incubated with the primary antibody at 4°C overnight. After 1 h of incubation at room temperature with the secondary antibody, the immunoblot signals were detected using the ultra‐sensitive electrochemiluminescence kit (Beyotime, China) on the ChemiScope6000 system (Clinx, China). Rabbit anti‐human primary antibodies (Anti‐SOX2, Anti‐Oct‐4, Anti‐CD44, Anti‐CD133, Anti‐HK2, Anti‐LDHA, Anti‐β‐catenin, Anti‐AKT, and Anti‐β‐actin) and goat anti‐rabbit IgG H&L (HRP) were obtained from Abcam (UK).^19^ Anti‐p‐AKT was obtained from Cell Signaling Technology (USA).

### 2.10 Extracellular acidification rate and oxygen consumption rate

The Seahorse XFe 96 extracellular flux analyzer (Seahorse, USA) was utilized to determine glycolysis and oxidative phosphorylation. Cells were seeded in Seahorse XFe 96 cell culture plates with 12,000 cells per well to achieve 90% confluence. The procedures were followed when using the Seahorse XF glycolysis stress test and Seahorse XF mitochondrial stress test reagent kits. These cells were transferred from a culture medium to an assay medium just before the assays, and they were incubated for 1 h at 37°C. Following baseline measurements, different chemicals prepared in the assay medium were injected into each well in turn, and the extracellular acidification rate (ECAR) or oxygen consumption rate (OCR) was calculated as a result. Glucose, oligomycin, or 2‐deoxy‐D‐glucose (2‐DG) should be added at the designated times for the ECAR measurement. Rotenone, carbonyl cyanide 4‐(trifluoromethoxy)phenylhydrazone (FCCP), oligomycin, or antimycin A should be added for the OCR at the designated times.^21^

### 2.11 Lactate production

The Lactate (LA) assay kit (Solarbio, China) was used to measure LA production following the instructions.^21^

### 2.12 Dual‐luciferase reporter assay

pGL3‐STIL‐promoter‐WT and pGL3‐STIL‐promoter‐MUT luciferase reporter vectors (Promega, USA) were developed to examine the binding relationship between transcription factor RFX5 and STIL. Plasmid sh‐negative control (NC) or sh‐RFX5 was co‐transfected into 293T cells. After 48 h of incubation, the luciferase activity was measured using the luciferase activity assay kit (Promega, USA).^16^

### 2.13 Chromatin immunoprecipitation

Chromatin immunoprecipitation (ChIP) assays were carried out using IP‐grade anti‐lgG antibodies (Abcam, UK) and Anti‐RFX5 antibodies (Thermo Fisher Scientific, USA) under Kit's (Merck, Germany) instructions.^22^ qPCR was used to detect purified DNA, and the primers used are displayed in Table 2.

**TABLE 2:** Primers used in chromatin immunoprecipitation quantitative real‐time polymerase chain reaction.

Primer	Sequence	
Site	Forward primer	5′‐ACCCACTGATAGAGGGCTGA‐3′
	Reverse primer	5′‐CCTGTTGAGTAGAAGAAAAGAATGT‐3’

### 2.14 Construction of a mouse model of xenograft tumors

In order to investigate the effect of RFX5/STIL axis on tumor progression in mice in vivo, we purchased 15 BALB/c nude mice around 6 weeks of age from Hangsi Biotechnology Co. Ltd. (Hangzhou, China) and subcutaneously injected SKHEP‐1 cells transfected with sh‐NC/sh‐RFX5 or oe‐NC/oe‐STIL, respectively, into nude mice and constructed the following groupings: sh‐NC + oe‐NC, sh‐RFX5 + oe‐NC, and sh‐RFX5 + oe‐STIL. The size of the tumor volume, the longest diameter of the tumor (*L*) and the longest transverse meridian of the tumor in the vertical direction (*W*) were recorded at 5‐day intervals since the inoculation of SKHEP‐1 cells and were calculated according to the formula Volume = (*L* × *W* ^2^)/2. After the 25th day, the mice were euthanized, and the tumor tissues were removed from the body to be weighed and the data recorded. The above steps of animal experiments were performed in strict accordance with the requirements of Anyang Tumor Hospital Laboratory Animal Ethics Committee.

Tumor tissues in the above different treatment groups were collected for immunohistochemistry (IHC) experiments in Section 2.3 and Western blot (WB) experiments in Section 2.9.

### 2.15 Statistical analysis

Data are presented as mean ± standard deviation. GraphPad 8.0 was used for *t*‐test or one‐way analysis of variance to evaluate the data, and the comparison of differences between several groups was performed by multivariate regression analysis. Statistical significance was defined as the *p* value of less than 0.05.

## 3 RESULTS

### 3.1 Upregulated STIL in HCC

**FIGURE 1:**
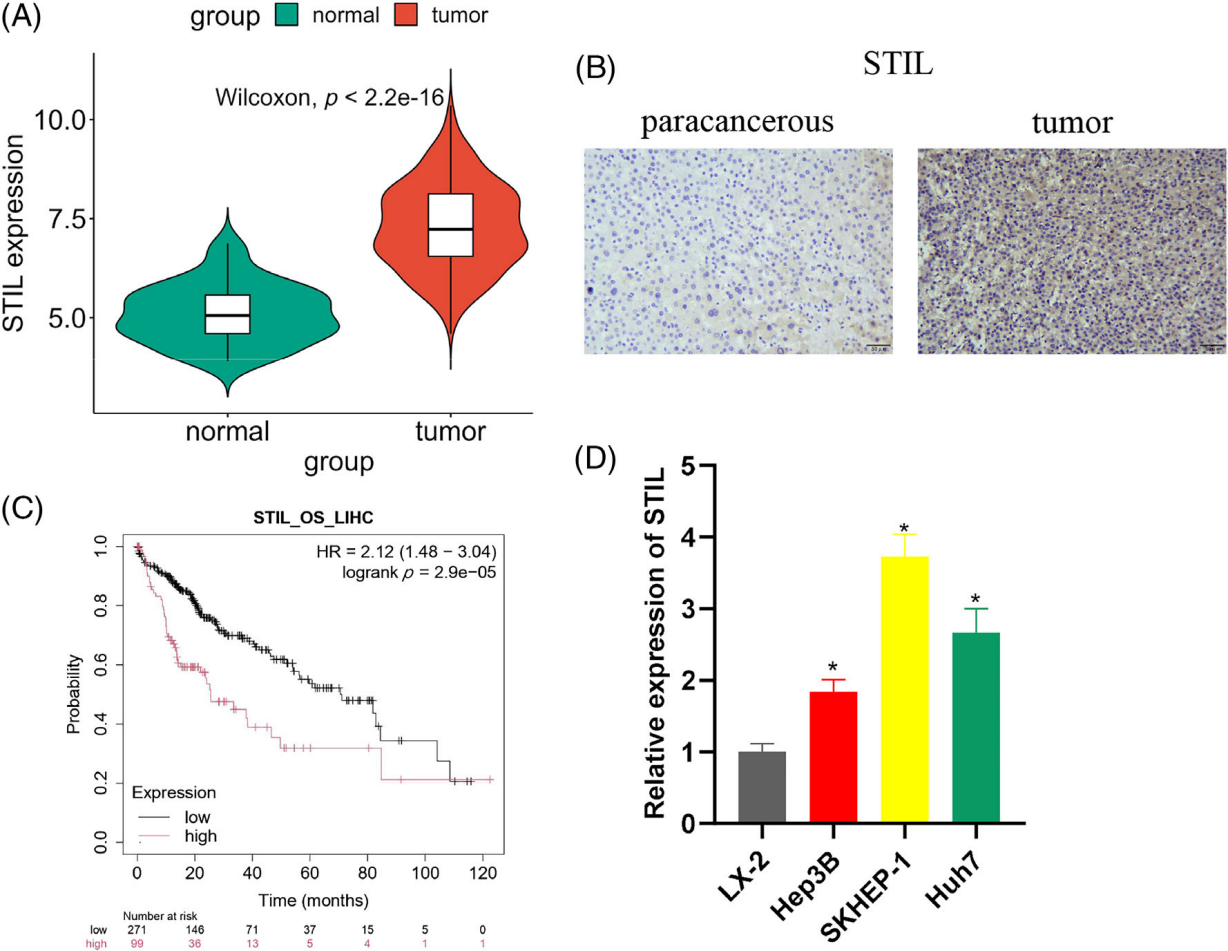
The upregulation of SCL/TAL1 interruption site (STIL) in hepatocellular carcinoma (HCC). (A) Analysis of *STIL* expression in HCC tissues using The Cancer Genome Atlas database. (B) Immunohistochemistry analysis of STIL expression in HCC tissues. (C) Kaplan–Meier survival curve analysis of the correlation between *STIL* and HCC patients' prognoses. (D) *STIL* expression in human normal liver cells and human HCC cells. * indicates *p* < 0.05.

We performed bioinformatics analysis on the TCGA database to analyze differential expression of mRNAs and obtained 4860 differential mRNAs (3800 upregulated, 1060 downregulated). Then we used the Wilcoxon test to find the significantly high expression of *STIL* in HCC patients (Figure 1A). IHC analysis showed that STIL was significantly highly expressed in HCC tissues (Figure 1B). Kaplan–Meier analysis showed that high *STIL* expression was associated with poor prognosis in HCC patients (Figure 1C). Furthermore, the *STIL* expression was considerably upregulated in human liver cancer cell lines as opposed to normal liver cells. Hep3B had the lowest STIL expression among the cell lines, while SKHEP‐1 had the highest (Figure 1D). The above results indicated that STIL was upregulated in HCC.

### 3.2 HCC cells stemness regulated by STIL

**FIGURE 2:**
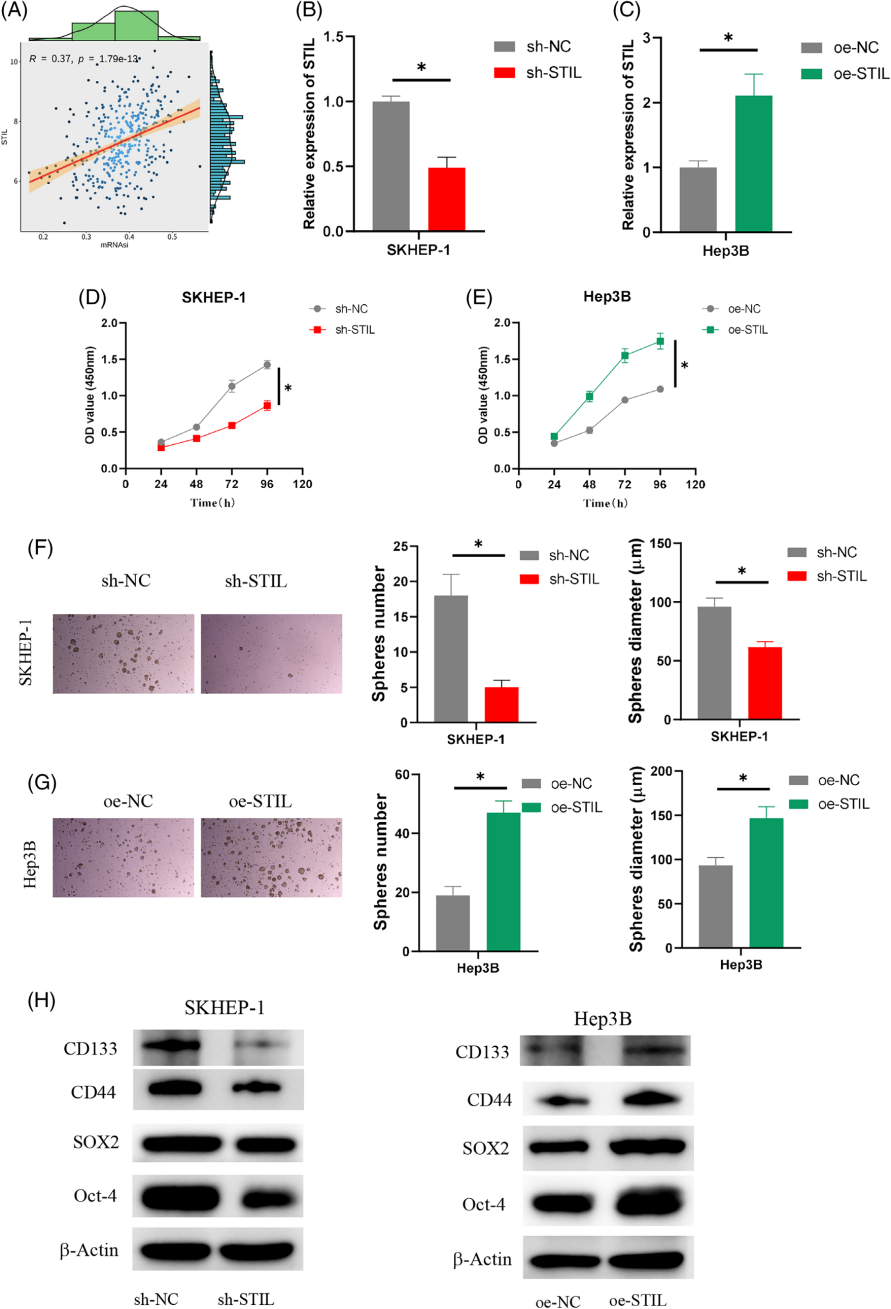
The effect of SCL/TAL1 interruption site (STIL) on the hepatocellular carcinoma (HCC) cell stemness. (A) Correlation analysis of stemness index mRNAsi and *STIL*. (B, C) Determination of transfection efficiency. (D, E) Determination of cell viability. (F, G) Determination of cell sphere formation ability. (H) Determination of SOX2, Oct‐4, CD133, and CD44 expression. * indicates *p* < 0.05. OD: optical density.

Bioinformatics analysis revealed a positive correlation with a value of 0.37 for mRNA expression‐based Stemness Index (mRNAsi) negative control between *STIL* and HCC (Figure 2A). We developed two cell groups with STIL overexpression and STIL knockdown treatment, respectively, to examine the possible function of STIL in the HCC cell stemness. qRT‐PCR was conducted to assess the *STIL* expression in SKHEP‐1 and Hep3B cells. In SKHEP‐1 cells, *STIL* expression was downregulated upon STIL knockdown, whereas STIL overexpression resulted in an increase in *STIL* expression in Hep3B cells (Figure 2B,C). The CCK‐8 assay demonstrated that SKHEP‐1 cell viability decreased when STIL was knocked down (Figure 2D), while overexpression of STIL promoted the viability of Hep3B cells (Figure 2E). The ability of cell sphere formation was detected by the sphere formation assay. The findings revealed that sh‐STIL strongly prevented the SKHEP‐1 cell sphere from forming (Figure 2F), while oe‐STIL enhanced the formation of Hep3B cell spheres (Figure 2G). The expression of the stem cell markers Oct‐4, SOX2, CD133, and CD44 was measured by WB analysis.^23^, ^24^ The results indicated that overexpression of STIL enhanced the SOX2, Oct‐4, CD133, and CD44 expression in Hep3B cells while silencing STIL could inhibit the SOX2, Oct‐4, CD133, and CD44 expression in SKHEP‐1 cells (Figure 2H). In conclusion, our results revealed that STIL enhanced HCC cell stemness.

### 3.3 The impact of STIL on the HCC cell stemness through aerobic glycolysis

**FIGURE 3:**
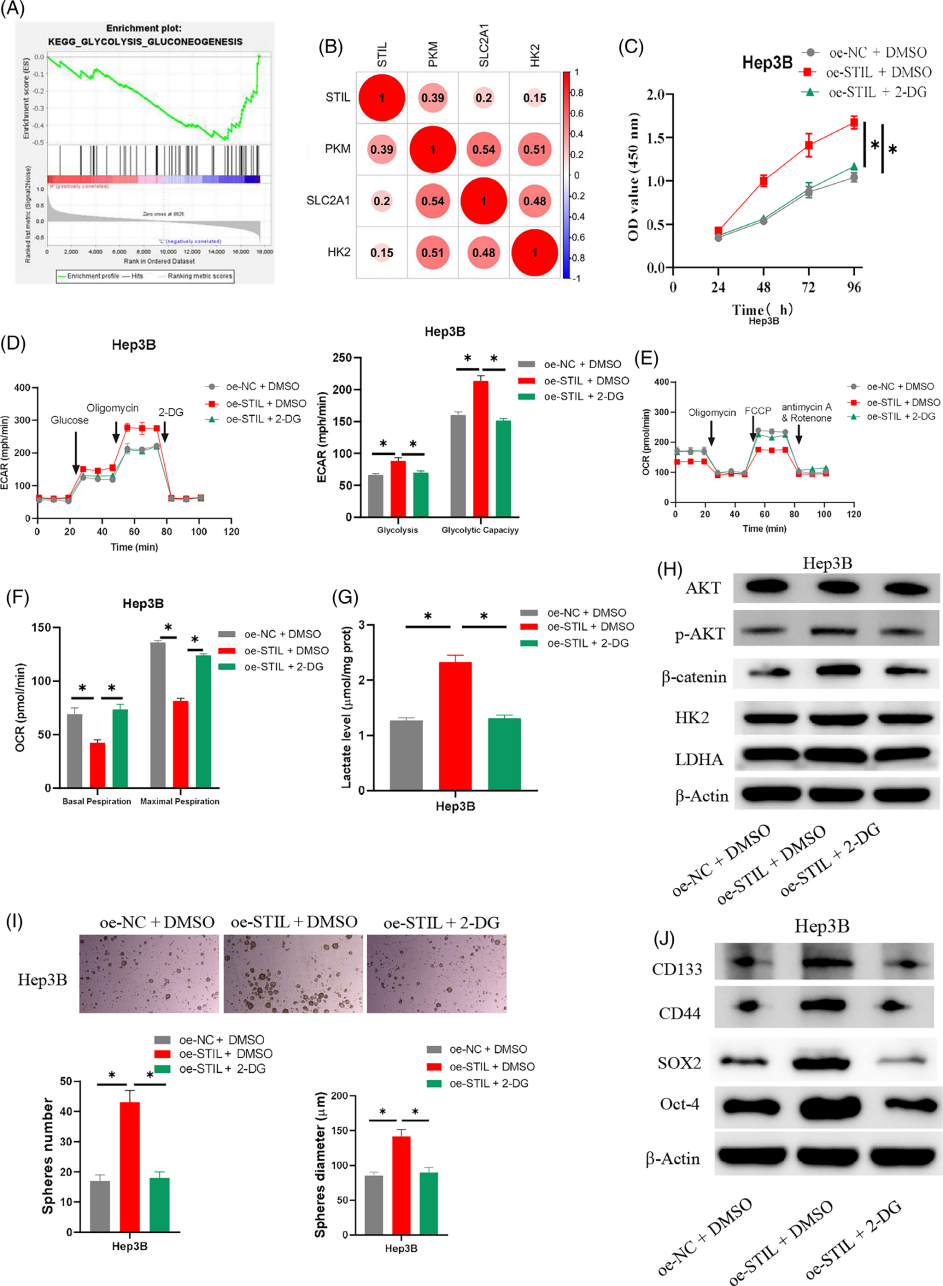
Regulatory mechanism of SCL/TAL1 interruption site (STIL) in regulating stemness of hepatocellular carcinoma (HCC) cells. (A) Enrichment pathway analysis of *STIL*. (B) Correlation analysis of *STIL* and glycolysis pathway markers. (C) Determination of cell viability. (D–F) Detection of extracellular acidification rate (ECAR) and oxygen consumption rate (OCR) in HCC cells. (G) Detection of the lactate production in HCC cells. (H) Detection of AKT, p‐AKT, β‐catenin, HK2, and LDHA expression. (I) Determination of cell sphere formation ability. (J) Detection of SOX2, Oct‐4, CD133, and CD44 expression. * indicates *p* < 0.05. 2‐DG, 2‐deoxy‐D‐glucose; FCCP, carbonyl cyanide 4‐(trifluoromethoxy)phenylhydrazone.

To further explore the possible pathways through which STIL affects the stemness of HCC cells, we carried out bioinformatics analysis and discovered that *STIL* was markedly enriched in the glycolysis pathway (Figure 3A). Correlation analysis indicated a positive correlation between *STIL* and markers of the glycolysis pathway solute carrier family 2 member 1 (*SLC2A1*), hexokinase 2 (*HK2*), and pyruvate kinase M (*PKM*) (Figure 3B). We established the cell groups oe‐NC + dimethyl sulfoxide (DMSO), oe‐STIL + DMSO, oe‐STIL + 2‐DG, sh‐NC + DMSO, sh‐STIL + DMSO, and sh‐STIL + glucose to further investigate the relationship between STIL and the stemness of HCC cells through glycolysis. Cell viability was assessed by CCK‐8 assay. The results indicated that remarkably upregulating STIL enhanced Hep3B cell viability, which was restored by 2‐DG treatment (Figure 3C). STIL knockdown expression significantly inhibited SKHEP‐1 cell viability, while glucose treatment was able to rescue the inhibitory effect (Figure S1A). The Seahorse assay was employed to detect ECAR and OCR to study the connection between STIL and metabolism. The results suggested that overexpressing STIL in HCC cells greatly enhanced glycolysis levels and capacity, but that the promoting effect of overexpressed STIL on ECAR was attenuated upon the addition of 2‐DG (Figure 3D). Glycolytic levels and glycolytic capacity were significantly reduced in STIL knockdown HCC cells, while further addition of glucose reverted the inhibitory effect of knockdown STIL on ECAR (Figure S1B,C). Overexpression of STIL significantly reduced the basal and maximal OCRs of HCC cells, and this inhibitory effect on OCR was weakened after further addition of 2‐DG (Figure 3E,F). Knockdown of STIL significantly elevated basal and maximal OCRs in SKHEP‐1 cells, while further addition of glucose reverted the promotion of OCR by knockdown of STIL (Figure S1D,E). The detection results of lactate production revealed that overexpression of STIL significantly increased lactate production in HCC cells relative to the control group, while the addition of 2‐DG in oe‐STIL‐treated cancer cells brought lactate production back to the level of the control group (Figure 3G). Knockdown of STIL significantly inhibited the production of lactate, a glycolytic metabolite, in SKHEP‐1 cells, while lactate production in the cells reverted to the control level after the addition of glucose treatment (Figure S1F). STIL can regulate the expression level of β‐catenin through phosphorylated AKT (p‐AKT).^15^ Furthermore, WB analysis of glycolysis pathway‐related proteins (HK2 and LDHA), AKT, p‐AKT, and β‐catenin suggested that STIL overexpression considerably promoted the expression of HK2, LDHA, p‐AKT, and β‐catenin in Hep3B cells, and the promoting effect was counteracted by the addition of 2‐DG (Figure 3H). STIL knockdown significantly inhibited the expression of HK2, LDHA, p‐AKT, and β‐catenin, while further addition of glucose treatment was able to reverse the inhibitory effect (Figure S1G). The stem cell sphere formation assay results demonstrated that oe‐STIL markedly improved Hep3B cells' capacity to form spheres, but that the promoting effect could be countered by further addition of 2‐DG (Figure 3I). STIL knockdown significantly reduced the ability of SKHEP‐1 cells to form spheroids, while further treatment with added glucose was able to reverse the inhibitory effect of knockdown of STIL on the ability of SKHEP‐1 cells to form spheroids (Figure S1H). The expression levels of stem cell markers (SOX2, Oct‐4, CD133, and CD44) were detected by WB. According to the results, overexpression of STIL in Hep3B HCC cells may enhance the expression of SOX2, Oct‐4, CD133, and CD44 compared to the control group. However, the addition of 2‐DG treatment may reverse the above‐mentioned promoting effect (Figure 3J). STIL knockdown reduced the expression of SOX2, Oct‐4, CD133, and CD44, important markers of stem cells, in SKHEP‐1 cells, while further addition of glucose treatment reversed the inhibitory effect (Figure S1I). To summarize, the experimental results suggested that STIL may act as a mediator in the glycolysis pathway to enhance the stemness of HCC cells.

### 3.4 Regulation of STIL expression by RFX5

**FIGURE 4:**
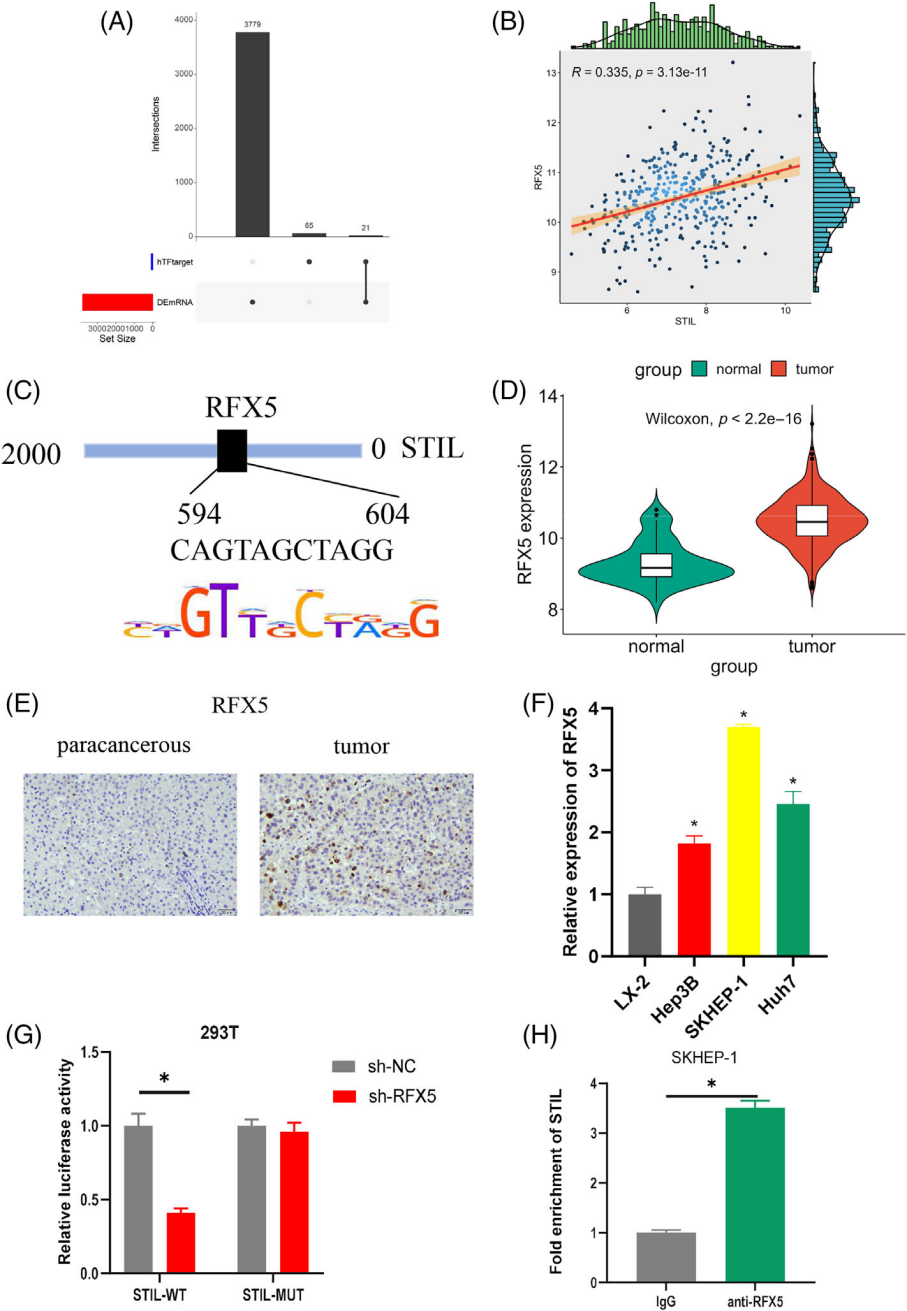
The regulatory role of RFX5 on SCL/TAL1 interruption site (STIL). (A) UpSet plot of predicted transcription factors and differentially expressed genes. (B) Scatter plot of the correlation between *RFX5* and *STIL*. (C) The binding sites of *STIL* and *RFX5*. (D) Analysis of *RFX5* expression in hepatocellular carcinoma (HCC) tumor tissues and adjacent cancerous tissues. (E) Immunohistochemistry analysis of RFX5 expression in HCC tissues. (F) The *RFX5* expression in HCC cells and human normal liver cells. (G, H) Validation of the binding relationship between RFX5 and STIL. * indicates *p* < 0.05. 2‐DG, 2‐deoxy‐D‐glucose; ECAR, extracellular acidification rate; FCCP, carbonyl cyanide 4‐(trifluoromethoxy)phenylhydrazone; OCR, oxygen consumption rate.

To find the potential molecular mechanisms of STIL in regulating HCC cells, we used KnockTF database to predict the potential upstream transcription factor of STIL. Eighty‐six potential transcription factors were obtained, which were intersected with upregulated differential mRNAs to obtain 21 potential transcription factors (Figure 4A). *STIL* and *RFX5* were found to have a positive correlation of 0.335 by Pearson correlation analysis (Figure 4B). Several putative binding sites between *STIL* and the promoter region of *RFX5* were discovered via the prediction from the HOCOMOCO database (Figure 4C). *RFX5* was found to be significantly overexpressed in HCC tumor tissues by bioinformatics analysis (Figure 4D). IHC analysis showed that RFX5 was significantly overexpressed in HCC tissues (Figure 4E). qRT‐PCR detected the expression of *RFX5* in HCC cells and human normal liver cells. The results indicated that *RFX5* was highly expressed in HCC cells (Figure 4F). The results of dual‐luciferase assay and ChIP experiments suggested that, in RFX5‐silenced 293T cells, the luciferase activity of STIL‐WT was greatly decreased, whereas in the STIL‐MUT group, there was no clear difference. Anti‐RFX5 significantly increased the enrichment of STIL (Figure 4G,H). These results indicated that transcription factor RFX5 could target and regulate STIL.

### 3.5 RFX5 activates STIL‐mediated regulation of aerobic glycolysis to promote HCC cell stemness

**FIGURE 5:**
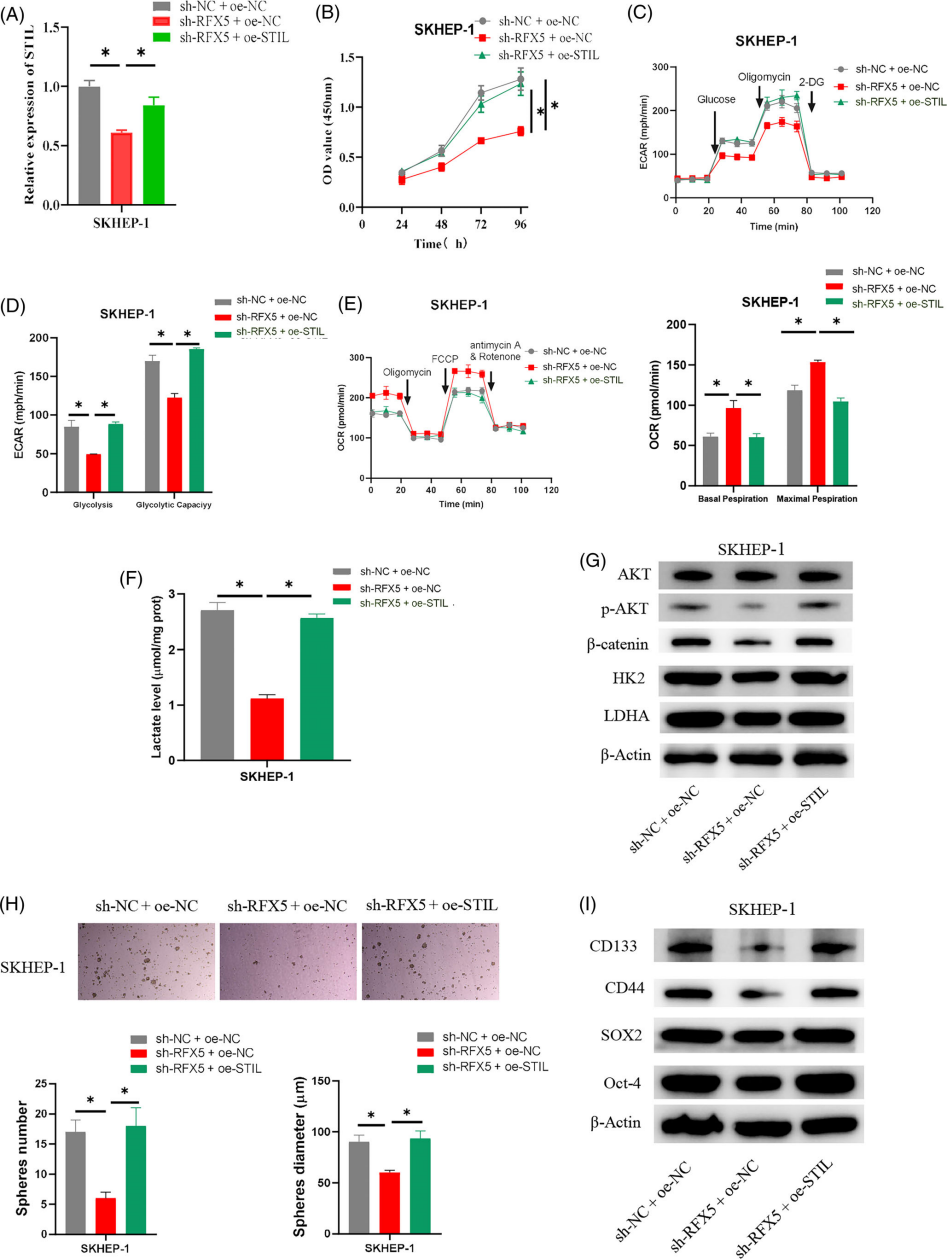
RFX5 activates SCL/TAL1 interruption site (STIL) to regulate aerobic glycolysis and promote hepatocellular carcinoma (HCC) stemness. (A) Quantitative real‐time polymerase chain reaction detection of transfection efficiency. (B) CCK‐8 detection of cell viability. (C–E) Seahorse XFe96 analysis of ECAR and OCR of HCC cells. (F) Determination of lactate production in HCC cells. (G) Western blot (WB) detection of expression of glycolytic metabolism pathway‐related proteins (HK2 and LDHA) AKT, p‐AKT, and β‐catenin. (H) Cell sphere formation assay detection of cell sphere‐forming ability. (I) WB analysis of the expression of stem cell surface markers (SOX2, Oct‐4, CD133, and CD44). * indicates *p* < 0.05. 2‐DG, 2‐deoxy‐D‐glucose; ECAR, extracellular acidification rate; OCR, oxygen consumption rate.

The groups namely sh‐NC + oe‐NC, sh‐RFX5 + oe‐NC, and sh‐RFX5 + oe‐STIL were constructed to examine the molecular mechanism through which RFX5 controls the function of STIL in HCC. The results of qRT‐PCR demonstrated that RFX5 knockdown greatly reduced *STIL* expression, but that STIL overexpression could counteract the inhibitory effects (Figure 5A). The CCK‐8 assay results evidenced that RFX5 knockdown markedly reduced cell viability, but that this effect could be reversed by further overexpressing STIL (Figure 5B). ECAR detection evidenced that SKHEP‐1 cells with RFX5 knockdown had significantly lower levels of glycolysis and glycolysis capacity, which could be reversed by further overexpressing STIL (Figure 5C,D). OCR results revealed that SKHEP‐1 cells with RFX5 knockdown had significantly higher basal and maximum OCRs. However, additional STIL overexpression could reverse the above‐promoting effects (Figure 5E). In SKHEP‐1 cells with knocked‐down RFX5, lactate production was inhibited, and further overexpression of STIL could undo the effect of RFX5 knockdown on lactate levels (Figure 5F). WB was utilized to determine the expression levels of specific genes (HK2 and LDHA) related to the glycolysis metabolism pathway. The results indicated that in HCC cells, RFX5 knockdown could suppress the expression of proteins that participated in the glycolysis metabolism pathway (HK2 and LDHA), p‐AKT, and β‐catenin, and additional STIL overexpression could counteract the inhibitory effect (Figure 5G). The sphere formation assay was performed to assess the sphere‐forming ability of stem cells. The findings revealed that SKHEP‐1 cells' capacity to form spheres could be weakened by knocking down RFX5, but that this effect could be reversed by further overexpressing STIL (Figure 5H). WB results showed that knocking down RFX5 significantly reduced the expression of stem cell surface marker proteins (SOX2, Oct‐4, CD133, and CD44), and further overexpression of STIL could reverse the effect (Figure 5I). The outcomes above confirmed that RFX5 may facilitate HCC cell stemness by stimulating the STIL transcription via the glycolysis pathway.

### 3.6 RFX5 activates STIL to promote HCC cell stemness in mice

**FIGURE 6:**
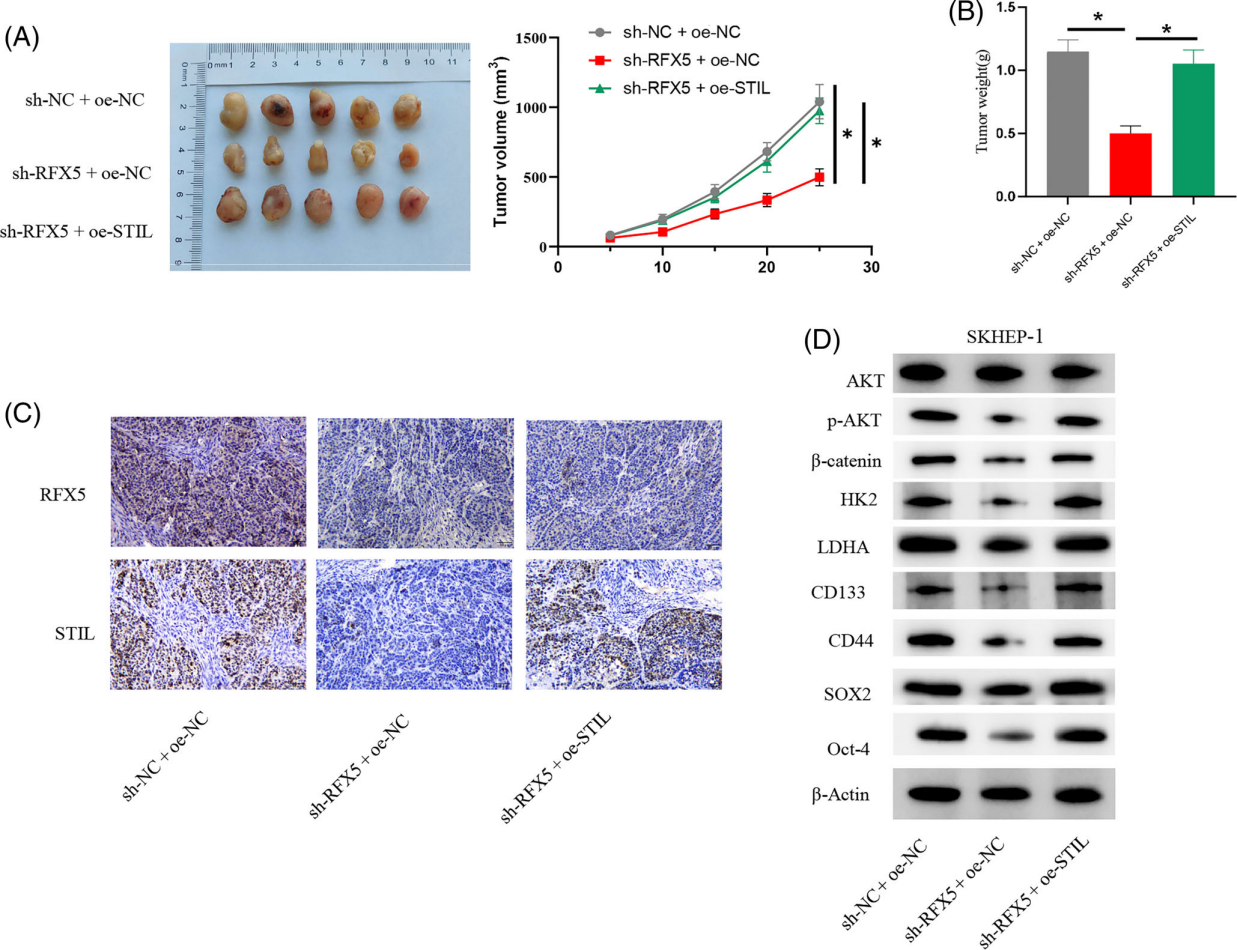
RFX5 activates SCL/TAL1 interruption site (STIL) to promote stemness of hepatocellular carcinoma (HCC) cells in mice through glycolytic pathway. (A, B) Changes in tumor size as well as mass in mice. (C) Immunohistochemistry detection of RFX5 and STIL protein expression in HCC tissues. (D) Western blot analysis of the expression of stem cell surface markers (SOX2, Oct‐4, CD133, and CD44), glycolytic metabolism pathway‐related proteins (HK2 and LDHA), AKT, p‐AKT, and β‐catenin. * indicates *p* < 0.05. FCCP, carbonyl cyanide 4‐(trifluoromethoxy)phenylhydrazone.

The above experiments have confirmed that RFX5 activation promotes aerobic glycolysis through STIL regulation to enhance the stemness of HCC cells. To investigate the application of this molecular mechanism in mice, we also established the following groups: sh‐NC + oe‐NC; sh‐RFX5 + oe‐NC; sh‐RFX5 + oe‐STIL. The experimental results showed that knocking down RFX5 significantly inhibited the volume and mass of HCC tumors in mice, while further overexpression of STIL reversed the inhibitory effect (Figure 6A,B). IHC experimental results indicated that knocking down RFX5 significantly inhibited the protein expression of RFX5 and STIL in tumor tissues, while further overexpression of STIL reversed the inhibitory effect of RFX5 knockdown on STIL protein expression without affecting RFX5 protein expression (Figure 6C). WB experimental results showed that knocking down RFX5 significantly reduced the protein expression of stem cell markers (CD133, CD44, SOX2, and Oct‐4) and proteins related to the glycolysis pathway (HK2 and LDHA), as well as the expression of p‐AKT and β‐catenin in tumor tissues, while overexpression of STIL could reverse the inhibitory effect of RFX5 knockdown on protein expression (Figure 6D). The above experiments confirmed that RFX5 activation promoted the stemness of HCC cells in mice through STIL.

## 4 DISCUSSION

HCC is the most common histological subtype of liver cancer, making up about 80%–90% of cases, and the incidence of liver cancer is still on the rise.^25^ HCC is also one of the most aggressive malignancies.^26^ Recent advances have indicated the presence of CSCs in liver cancer.^27^ The progression of HCC includes the gradual loss of differentiated phenotype and the acquisition of stemness features.^28^ Stemness in cancer cells is the primary cause of tumor metastasis, recurrence, and chemoresistance, which are the main obstacles in tumor treatment.^29^ It is still difficult to find strategies that will effectively eradicate CSCs. Consequently, it is imperative to investigate the molecular regulatory mechanisms that affect the stemness of HCC to develop more effective therapeutic strategies for HCC.

STIL is a cell cycle regulatory protein that is specifically recruited to the centrosome during mitosis to enhance centriole duplication in dividing cells.^9^ There has been accumulating evidence that many types of tumors have high expression levels of STIL, including lung cancer, where its expression is markedly elevated. Silencing STIL can inhibit tumor metastasis and growth, while overexpression of STIL triggers the epithelial‐to‐mesenchymal transition (EMT) pathway, enhancing cancer cell invasion and migration.^30^ Yu et al.^31^ reported that the phosphatidylinositol 3‐kinas (PI3K)/protein kinase B (AKT)/mammalian target of rapamycin (mTOR) signaling pathway can be promoted by STIL, which can raise c‐myc expression and ultimately encourage the occurrence and development of bladder cancer. Furthermore, high expression of STIL in colorectal cancer has been observed. Knocking down STIL expression can significantly inhibit the protein expression of stem cell markers CD133 and CD44 by downregulating β‐catenin levels through the inhibition of p‐AKT expression.^15^ In this research, STIL was found to be considerably elevated in HCC cells and tissues. Because the tumor progression is attributed to the presence of CSCs, studies have proved that STIL is involved in regulating cancer cell stemness. For example, STIL promotes colorectal cancer cell proliferation and tumor growth by enhancing Shhh and Wnt signaling pathways and stimulates stemness in tumor cells.^15^ Nevertheless, the role of STIL in HCC stemness has not been verified. Therefore, we examined the relationship between the STIL expression and the stemness index of HCC and found that STIL was positively correlated with the mRNAsi of HCC. Additional cellular functional experiments revealed that STIL could greatly enhance stemness, indicating the involvement of STIL in regulating HCC cell stemness. The results have contributed to the inhibition of HCC stemness by targeting STIL.

Furthermore, to investigate the connection between STIL and the stemness of HCC cells, we discovered via enrichment pathway analysis that STIL was enriched in the HCC glycolysis signaling pathway. Reports have stated that aerobic glycolysis contributes substantially to the development of HCC, and phosphofructokinase 1 in glycolysis can stimulate the HCC cell proliferation and preserve the stemness of cancer cells.^32^ SNRPB activates the AKT pathway in HCC cells by promoting the production of splice variants LDHA‐220 and AKT3‐204, stimulating cell proliferation and stemness of HCC.^33^ However, whether STIL could preserve the stemness of HCC cells through glycolysis remains unknown. We discovered that STIL may enhance tumor stemness by stimulating the glycolysis pathway of HCC cells through cell functional experiments. Here, we screened and validated RFX5 as a key transcription factor of STIL in HCC. RFX5 is a DNA‐binding regulatory factor, consisting of the transcription factor complex RFX and two other subunits, RFXAP and RFXANK/B.^34^, ^35^ Previous studies have shown that RFX5 is involved in the occurrence and development of several human tumors, such as stomach adenocarcinoma and non‐small cell lung cancer.^36^, ^37^ For example, RFX5 can stimulate the progression of breast cancer cells by transcriptionally activating LINC00504 to reduce the expression of miR‐140‐5p.^38^ In HCC, RFX5 binds to the promoter region of KDM4A to upregulate the expression of KDM4A, thereby inhibiting tumor apoptosis and promoting cell cycle progression.^34^ In this study, we found that RFX5 was highly expressed in HCC and could activate STIL expression by regulating glycolysis to promote cancer cell stemness. These findings clarified STIL's function in the development of HCC and its upstream regulatory mechanism. Our study revealed a new mechanism for the RFX5/STIL axis in HCC cell stemness.

In summary, we confirmed that the RFX5/STIL axis promoted HCC cell stemness by regulating aerobic glycolysis, suggesting that RFX5/STIL may serve as potential biomarkers in HCC. However, there are still certain limitations in this study, such as the lack of clinical and animal validation for the effects of RFX5/STIL on the HCC cells stemness. Therefore, future research is planned to examine the functional mechanism of RFX5/STIL in influencing the HCC stemness and offering guidelines and treatment approaches for the HCC. Our study contributes to a better understanding of the development and regulatory mechanisms of HCC.

## CONFLICT OF INTEREST STATEMENT

The authors declare no conflict of interest.

## ETHICS STATEMENT

This study was approved by Anyang Tumor Hospital Ethical Review Committee, approval no. 2024DW05K01.

## Supporting information


**FIGURE S1.** Regulatory mechanism of STIL in regulating stemness of HCC cells. (A) Determination of cell viability. (B–E) Detection of ECAR and OCR in HCC cells. (F) Detection of the lactate production in HCC cells. (G) Detection of AKT, p‐AKT, β‐catenin, HK2, and LDHA expression. (H) Determination of cell sphere formation ability. (I) Detection of SOX2, Oct‐4, CD133, and CD44 expression. * indicates *p* < 0.05.

## Data Availability

The data and materials in the current study are available from the corresponding author on reasonable request.

## References

[1] Samant H , Amiri HS , Zibari GB . Addressing the worldwide hepatocellular carcinoma: epidemiology, prevention and management. J Gastrointest Oncol. 2021;12(Suppl 2):S361–S373. 34422400 10.21037/jgo.2020.02.08 PMC8343080

[2] Deng S , Solinas A , Calvisi DF . Cabozantinib for HCC treatment, from clinical back to experimental models. Front Oncol. 2021;11:756672. 34722310 10.3389/fonc.2021.756672 PMC8548824

[3] Chakraborty E , Sarkar D . Emerging therapies for hepatocellular carcinoma (HCC). Cancers. 2022;14(11):2798. 35681776 10.3390/cancers14112798 PMC9179883

[4] Najafi M , Farhood B , Mortezaee K . Cancer stem cells (CSCs) in cancer progression and therapy. J Cell Physiol. 2019;234(6):8381–8395. 30417375 10.1002/jcp.27740

[5] Wang X , Wang J , Tsui YM , Shi C , Wang Y , Zhang X , et al. RALYL increases hepatocellular carcinoma stemness by sustaining the mRNA stability of TGF‐beta2. Nat Commun. 2021;12(1):1518. 33750796 10.1038/s41467-021-21828-7 PMC7943813

[6] Wang J , Zhou Y , Zhang D , Zhao W , Lu Y , Liu C , et al. CRIP1 suppresses BBOX1‐mediated carnitine metabolism to promote stemness in hepatocellular carcinoma. EMBO J. 2022;41(15):e110218. 35775648 10.15252/embj.2021110218 PMC9340481

[7] Bi L , Ren Y , Feng M , Meng P , Wang Q , Chen W , et al. HDAC11 regulates glycolysis through the LKB1/AMPK signaling pathway to maintain hepatocellular carcinoma stemness. Cancer Res. 2021;81(8):2015–2028. 33602787 10.1158/0008-5472.CAN-20-3044

[8] Chen YY , Wang WH , Che L , Lan Y , Zhang LY , Zhan DL , et al. BNIP3L‐dependent mitophagy promotes HBx‐induced cancer stemness of hepatocellular carcinoma cells via glycolysis metabolism reprogramming. Cancers. 2020;12(3):655. 32168902 10.3390/cancers12030655 PMC7139741

[9] Patwardhan D , Mani S , Passemard S , Gressens P , El Ghouzzi V . STIL balancing primary microcephaly and cancer. Cell Death Dis. 2018;9(2):65. 29352115 10.1038/s41419-017-0101-9 PMC5833631

[10] Li J , Yang Z , Qi Y , Liu X , Liu Y , Gao X , et al. STIL acts as an oncogenetic driver in a primary cilia‐dependent manner in human cancer. Front Cell Dev Biol. 2022;10:804419. 35155425 10.3389/fcell.2022.804419 PMC8826476

[11] Song H , Zhao H , Chen C , Zhang D , Wang X , He J . Elevated STIL predicts poor prognosis in patients with hepatocellular carcinoma. Medicine. 2023;102(7):e33004. 36800576 10.1097/MD.0000000000033004 PMC9936050

[12] Xu L , Zhang S , Feng J , Tan D , Sun H , Guo H . ncRNAs‐mediated overexpression of STIL predict unfavorable prognosis and correlated with the efficacy of immunotherapy of hepatocellular carcinoma. Cancer Cell Int. 2023;23(1):44. 36899391 10.1186/s12935-023-02869-y PMC10007768

[13] Ouyang Y , Jin YB , Chen XP , Zhang GY , Mao SL , Ling F , et al. STIL is upregulated in nasopharyngeal carcinoma tissues and promotes nasopharyngeal carcinoma proliferation, migration and invasion. Neoplasma. 2020;67(1):37–45. 31607137 10.4149/neo_2019_190306N192

[14] Ji SF , Wen SL , Sun Y , Huang PW , Wu H , He ML . The biological function and clinical significance of STIL in osteosarcoma. Cancer Cell Int. 2021;21(1):218. 33858425 10.1186/s12935-021-01922-y PMC8051131

[15] Pradhan T , Kumar V , Surya HE , Krishna R , John S , Jissa VT , et al. STIL endows oncogenic and stem‐like attributes to colorectal cancer plausibly by shh and Wnt signaling. Front Oncol. 2021;11:581671. 34485108 10.3389/fonc.2021.581671 PMC8416176

[16] Lin X , Li AM , Li YH , Luo RC , Zou YJ , Liu YY , et al. Silencing MYH9 blocks HBx‐induced GSK3beta ubiquitination and degradation to inhibit tumor stemness in hepatocellular carcinoma. Signal Transduct Target Ther. 2020;5(1):13. 32296025 10.1038/s41392-020-0111-4 PMC7018736

[17] Liu X , Gao J , Sun Y , Zhang F , Guo W , Zhang S . Clotrimazole inhibits HCC migration and invasion by modulating the ERK‐p65 signaling pathway. Drug des Devel Ther. 2022;16:863–871. 10.2147/DDDT.S354205 PMC8976522 35378926

[18] Lin X , Luo L , Zou Y , Chen J . Cancer stemness‐associated LINC02475 serves as a novel biomarker for diagnosis and prognosis prediction of hepatocellular carcinoma. Front Genet. 2022;13:991936. 36118852 10.3389/fgene.2022.991936 PMC9479154

[19] Jiang T , Yang J , Yang H , Chen W , Ji K , Xu Y , et al. SLC35B4 stabilizes c‐MYC protein by O‐GlcNAcylation in HCC. Front Pharmacol. 2022;13:851089. 35308201 10.3389/fphar.2022.851089 PMC8924407

[20] Zhou X , Luo J , Xie H , Wei Z , Li T , Liu J , et al. MCM2 promotes the stemness and sorafenib resistance of hepatocellular carcinoma cells via hippo signaling. Cell Death Discov. 2022;8(1):418. 36243809 10.1038/s41420-022-01201-3 PMC9569387

[21] Zhou Y , Lin F , Wan T , Chen A , Wang H , Jiang B , et al. ZEB1 enhances Warburg effect to facilitate tumorigenesis and metastasis of HCC by transcriptionally activating PFKM. Theranostics. 2021;11(12):5926–5938. 33897890 10.7150/thno.56490 PMC8058737

[22] Jiang L , Wang X , Ma F , Wang X , Shi M , Yan Q , et al. PITX2C increases the stemness features of hepatocellular carcinoma cells by up‐regulating key developmental factors in liver progenitor. J Exp Clin Cancer Res. 2022;41(1):211. 35765089 10.1186/s13046-022-02424-z PMC9238105

[23] Verma P , Shukla N , Kumari S , Ansari MS , Gautam NK , Patel GK . Cancer stem cell in prostate cancer progression, metastasis and therapy resistance. Biochim Biophys Acta Rev Cancer. 2023;1878(3):188887. 36997008 10.1016/j.bbcan.2023.188887

[24] Jeng KS , Chang CF , Sheen IS , Jeng CJ , Wang CH . Cellular and molecular biology of cancer stem cells of hepatocellular carcinoma. Int J Mol Sci. 2023;24(2):1417. 36674932 10.3390/ijms24021417 PMC9861908

[25] Khanam A , Kottilil S . New therapeutics for HCC: does tumor immune microenvironment matter? Int J Mol Sci. 2022;24(1):437. 36613878 10.3390/ijms24010437 PMC9820509

[26] Forner A , Reig M , Bruix J . Hepatocellular carcinoma. Lancet. 2018;391(10127):1301–1314. 29307467 10.1016/S0140-6736(18)30010-2

[27] Fang X , Yan Q , Liu S , Guan XY . Cancer stem cells in hepatocellular carcinoma: intrinsic and extrinsic molecular mechanisms in stemness regulation. Int J Mol Sci. 2022;23(20):12327. 36293184 10.3390/ijms232012327 PMC9604119

[28] Malta TM , Sokolov A , Gentles AJ , Burzykowski T , Poisson L , Weinstein JN , et al. Machine learning identifies stemness features associated with oncogenic dedifferentiation. Cell. 2018;173(2):338–354.e15. 29625051 10.1016/j.cell.2018.03.034 PMC5902191

[29] Tsui YM , Chan LK , Ng IO . Cancer stemness in hepatocellular carcinoma: mechanisms and translational potential. Br J Cancer. 2020;122(10):1428–1440. 32231294 10.1038/s41416-020-0823-9 PMC7217836

[30] Wang YW , Chen SC , Gu DL , Yeh YC , Tsai JJ , Yang KT , et al. A novel HIF1alpha‐STIL‐FOXM1 axis regulates tumor metastasis. J Biomed Sci. 2022;29(1):24. 35365182 10.1186/s12929-022-00807-0 PMC8973879

[31] Yu H , Chen L , Wang X , Tang F , Wan Z , Wang H , et al. STIL promotes tumorigenesis of bladder cancer by activating PI3K/AKT/mTOR signaling pathway and targeting C‐Myc. Cancers. 2022;14(23):5777. 36497260 10.3390/cancers14235777 PMC9739707

[32] Sha X , Wang K , Wang F , Zhang C , Yang L , Zhu X . Silencing PFKP restrains the stemness of hepatocellular carcinoma cells. Exp Cell Res. 2021;407(1):112789. 34418458 10.1016/j.yexcr.2021.112789

[33] Zhan YT , Li L , Zeng TT , Zhou NN , Guan XY , Li Y . SNRPB‐mediated RNA splicing drives tumor cell proliferation and stemness in hepatocellular carcinoma. Aging. 2020;13(1):537–554. 33289700 10.18632/aging.202164 PMC7834993

[34] Chen DB , Xie XW , Zhao YJ , Wang XY , Liao WJ , Chen P , et al. RFX5 promotes the progression of hepatocellular carcinoma through transcriptional activation of KDM4A. Sci Rep. 2020;10(1):14538. 32883983 10.1038/s41598-020-71403-1 PMC7471945

[35] Chen DB , Zhao YJ , Wang XY , Liao WJ , Chen P , Deng KJ , et al. Regulatory factor X5 promotes hepatocellular carcinoma progression by transactivating tyrosine 3‐monooxygenase/tryptophan 5‐monooxygenase activation protein theta and suppressing apoptosis. Chin Med J. 2019;132(13):1572–1581. 31188160 10.1097/CM9.0000000000000296 PMC6616235

[36] Guo L , Liu D . Identification of RFX5 as prognostic biomarker and associated with immune infiltration in stomach adenocarcinoma. Eur J Med Res. 2022;27(1):164. 36045400 10.1186/s40001-022-00794-w PMC9429337

[37] Yang Y , Li H , Liu Y , Chi C , Ni J , Lin X . MiR‐4319 hinders YAP expression to restrain non‐small cell lung cancer growth through regulation of LIN28‐mediated RFX5 stability. Biomed Pharmacother. 2019;115:108956. 31096145 10.1016/j.biopha.2019.108956

[38] Hou T , Ye L , Wu S . Knockdown of LINC00504 inhibits the proliferation and invasion of breast cancer via the downregulation of miR‐140‐5p. Onco Targets Ther. 2021;14:3991–4003. 34239305 10.2147/OTT.S294965 PMC8259944

