# Supplementary figures and images for: Regulatory factor X‐5/SCL/TAL1 interruption site axis promotes aerobic glycolysis and hepatocellular carcinoma cell stemness
Source: Kaohsiung J Med Sci. 2024 Dec 24;41(1):e12922. doi: 10.1002/kjm2.12922 (PMC11724169; doi:10.1002/kjm2.12922)

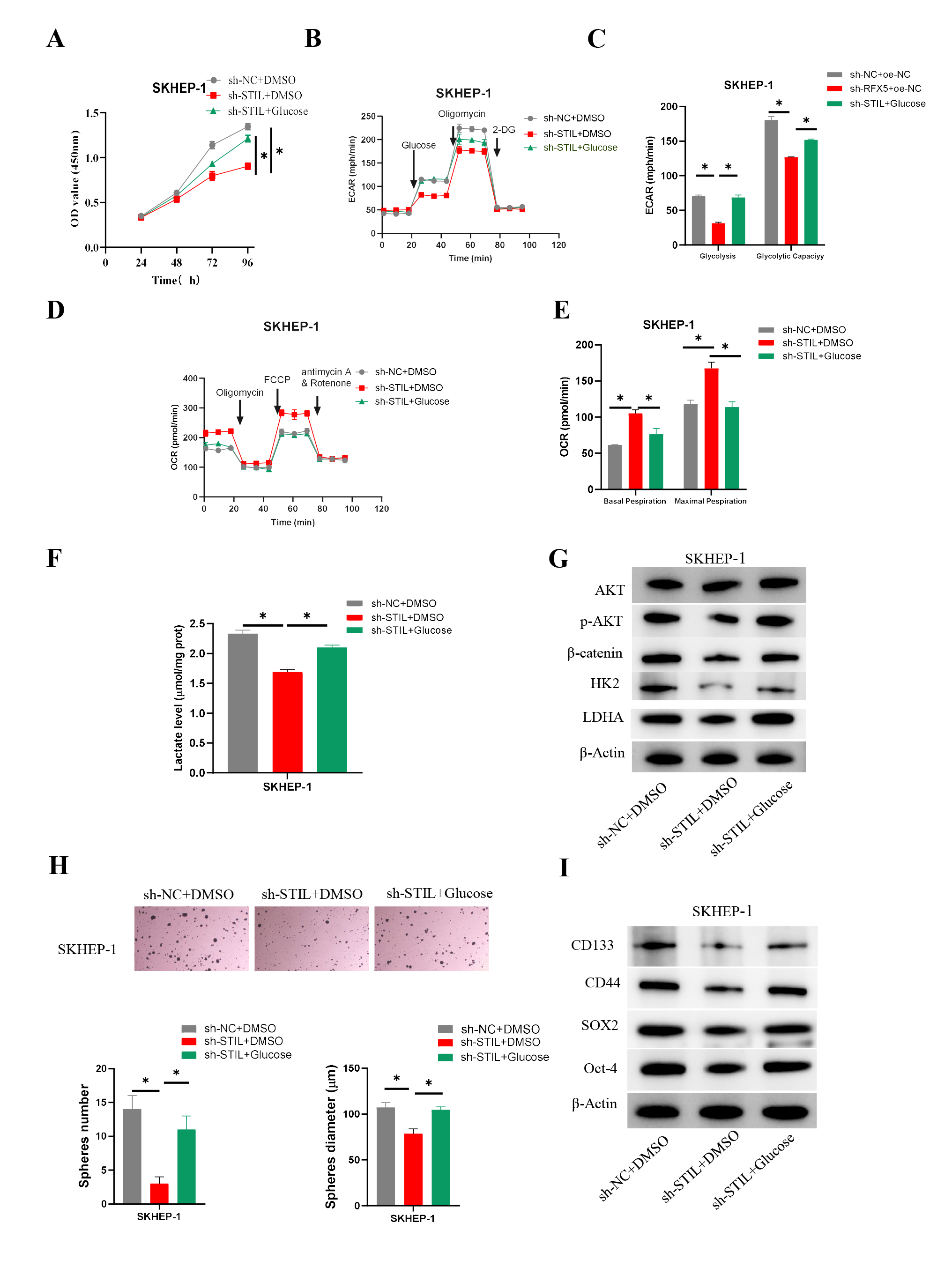

Supplement: Supplementary file 1 — FIGURE S1. Regulatory mechanism of STIL in regulating stemness of HCC cells. (A) Determination of cell viability. (B–E) Detection of ECAR and OCR in HCC cells. (F) Detection of the lactate production in HCC cells. (G) Detection of AKT, p‐AKT, β‐catenin, HK2, and LDHA expression. (H) Determination of cell sphere formation ability. (I) Detection of SOX2, Oct‐4, CD133, and CD44 expression. * indicates p < 0.05. [file KJM2-41-e12922-s001.tif]
